# Unveiling Nutritional Disparities in Infantile Tremor Syndrome: A Focus on Holo‐Transcobalamin and Essential Fatty Acids

**DOI:** 10.1155/jotm/3144439

**Published:** 2026-07-18

**Authors:** Rajesh Gupta, Puneet Agrawal, Jyoti Gupta, Jyoti Singh, Ashish Pathak

**Affiliations:** ^1^ Department of Pediatrics, Government Medical College, Datia, Madhya Pradesh, India, calicutmedicalcollege.ac.in; ^2^ Department of Microbiology, Mahaveer Institute of Medical Sciences, Bhopal, Madhya Pradesh, India; ^3^ Department of Pediatrics, Shyam Shah Medical College, Rewa, Madhya Pradesh, India; ^4^ Department of Pediatrics, R.D. Gardi Medical College, Ujjain, Madhya Pradesh, India, rdgmc.edu.in; ^5^ Health Systems and Policy (HSP): Medicines, Focusing Antibiotics, Department of Global Public Health, Karolinska Institutet, Stockholm, Sweden, ki.se

**Keywords:** clinical spectrum, developmental delay, essential fatty acids, holo-TC, holo-transcobalamin, infantile tremor syndrome, undernutrition, vitamin B12

## Abstract

Infantile tremor syndrome (ITS), a self‐limiting disorder prevalent in the Indian subcontinent, is characterized by tremors, anemia, pigmentary skin changes, developmental delay or regression, and hypotonia. Although a nutritional etiology is suspected, particularly vitamin B12 deficiency, the roles of the bioactive marker holo‐transcobalamin (holo‐TC) and essential fatty acids (EFAs) remain unexplored. This comparative pilot study was conducted from December 1, 2023, to April 30, 2024, at Government Medical College, Datia, India. The study included 14 infants who were assigned to two groups. Group 1 (*n* = 7) comprised infants with ITS and tremors, and Group 2 (*n* = 7) included those with ITS without tremors to assess threshold‐dependent biochemical markers across this clinical spectrum. Laboratory investigations included complete hemogram, serum vitamin B12, holo‐TC, and EFA profiles. Data were summarized using medians and interquartile ranges. Between‐group differences were expressed as effect sizes (median differences) with 95% confidence intervals. Severe developmental delays were more frequent in Group 1 (5/7) than in Group 2 (mild to moderate in 4/7). Both groups were anemic and had low serum vitamin B12 levels (median: 188 pg/mL in Group 1 vs. 198 pg/mL in Group 2). Holo‐TC was undetectable (< 5 pmol/L) in Group 1 and in only one patient from Group 2 (median: 10.12 pmol/L in the remaining Group 2 patients). Macrocytosis was higher in Group 1 (median MCV: 98 fL vs. 75.5 fL), with a median difference of 22.5 fL (95% CI: 6.8 to 38.2). Clinical examination also revealed characteristic hair hypochromotrichia and progressive regression in both phenotypes. Both groups exhibited low omega (*ω*)‐3 fatty acid levels, elevated saturated fatty acid levels, and high *ω*‐6:ω‐3 ratios (median: approximately 10:1), with small and imprecise between‐group differences (confidence intervals crossing zero). Extremely low holo‐TC levels were strongly associated with tremors in ITS, suggesting that holo‐TC is a superior marker of bioactive vitamin B12 deficiency and a critical metabolic threshold for tremor manifestation. EFA imbalances were prevalent in both groups but not specific to tremor incidence, highlighting the need for larger studies. The overall pathophysiology points toward significant maternal nutritional depletion during pregnancy and lactation as the relevant origin of the syndrome.

## 1. Introduction

Infantile tremor syndrome (ITS) is a self‐limiting clinical disorder in infants and young children, characterized by the presence of tremors, anemia, pigmentary skin disease, regression or delayed mental development, and hypotonia of muscles [1]. The reported incidence of ITS in pediatric hospital admissions is 0.2%–2% [1]. The disorder is reported predominantly in children from the Indian subcontinent [1].

The main etiology of ITS is nutritional [1]; however, the role of any specific nutrient in causation remains inconclusive. The diagnosis of ITS was reported exclusively in breast‐fed infants with a maternal diet restricted to vegetarian food and completely or partially lacking milk [2]. Infants diagnosed as having ITS are often those who are inadequately fed or lack complementary feeding [2]. In India, approximately 75% of the population has a vitamin B12 deficiency, which can be ascribed to the predominantly vegetarian diet [3]. Depending on maternal vitamin B12 levels, the body store of a normal newborn may last for eight months [4]. Multiple epidemiological, clinical, laboratory, and therapeutic studies have attempted to establish a causal association between vitamin B12 deficiency and ITS [1, 2]. Degeneration of nervous tissue has been reported in infants having vitamin B12 deficiency, where the maternal diet was a strict vegetarian diet devoid of milk or dairy products [3, 4]. Moreover, infants with ITS who were administered therapeutic doses of vitamin B12 displayed unequivocal improvements in their clinical condition and general well‐being within 48–72 h [2]. The aforementioned findings support the etiological association between vitamin B12 deficiency and ITS.

Vitamin B12 is an essential micronutrient for 1‐C metabolism and is derived from animal foods [5]. It functions as a coenzyme for methionine synthase and methylmalonyl CoA mutase [5]. Circulating vitamin B12 is bound to either of the carrier proteins, haptocorrin (HC) or transcobalamin (TC) [5]. Body cells can absorb only TC‐bound vitamin B‐12 (holo‐TC), which constitutes approximately 20% of the total, while the remaining 80% vitamin B‐12 exists in the HC‐bound form (holo‐HC) [6]. TC mediates the transport of vitamin B12 across the cell membrane after binding to the TC receptor and forming a protein–receptor complex, which is internalized by the lysosomes [6]. Lysosomal degradation of TC releases cobalamin, which remains inside the cells and undergoes further processing intracellularly [3]. Therefore, holo‐TC is considered a superior marker for acute changes in vitamin B12 homeostasis [3] as well as the bioactive form of vitamin B12 in plasma [3].

Essential fatty acids (EFAs), especially polyunsaturated fatty acids such as omega‐3 (ω‐3) and omega‐6 (ω‐6), cannot be produced in the body and require dietary sources. These fatty acids have been implicated in various neurodegenerative diseases in adults, with *ω*‐3 fatty acids reported to play crucial roles in the development and maintenance of normal CNS structure and function [7]. Humans have evolved consuming a diet containing nearly equal amounts of *ω-*3 and *ω-*6 EFAs; however, due to the increased intake of vegetable oils from corn, sunflower seeds, safflower seeds, cottonseeds, and soybeans, the *ω*‐6:*ω*‐3 fatty acid ratio in Western diets falls in the range 20–30:1 [7]. Lack of or inadequate complementary feeding can affect EFA levels, further influencing neuronal maturation in infants. However, very little is known about the role of EFAs in the nutrition of Indian children in health and disease. Therefore, the present study was planned as a pilot study to explore the roles of serum holo‐TC and EFA levels in the etiology of ITS among infants.

## 2. Material and Method

### 2.1. Study Design and Setting

This comparative study was conducted in the Department of Pediatrics, Government Medical College, Datia, M.P., India, from December 1, 2023, to April 30, 2024. The primary objective was to evaluate the roles of serum holo‐TC and EFAs in ITS etiology.

### 2.2. Participant Recruitment and Grouping

**FIGURE 1:**
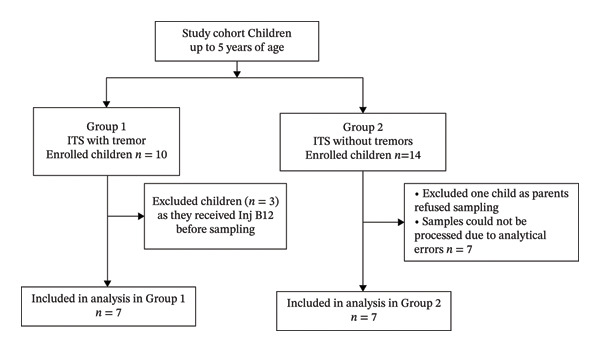
The recruitment process for participants included in the study.

The participants were enrolled based on the clinical case definition of ITS, which includes the presence of tremors, anemia, pigmentary skin disease, regression or delayed mental development, and muscular hypotonia [1]. The enrolled participants were assigned to either Group 1 (ITS with tremors) or Group 2 (ITS without tremors). The decision to group infants based on the presence or absence of tremors was primarily to explore whether holo‐transcobalamin (holo‐TC), as a bioactive marker, shows a specific association with this classic neurological manifestation, rather than to categorize infants based on overall clinical severity. Group 2 served as a comparison group, which comprised infants with similar clinical features but no active tremors. For participant selection, the study followed strict exclusion criteria, which are as follows: diagnosis at outside institutions, medical treatment history or any ongoing treatment, and history of vitamin B12 supplementation. Figure 1 shows the process of participant recruitment in the study.

### 2.3. Ethical Considerations

The research protocol received approval from the institutional ethics committee (approval number: 1219/Pedia/IECBMHR/GMC/2023/Version‐1). Prior to the enrollment of infants, written informed consent was obtained from their parents or legal guardians.

### 2.4. Data Collection and Clinical Assessment

A predesigned questionnaire was utilized to record comprehensive patient data, including demographic profiles, general and systemic examination findings, and developmental milestones. Developmental assessment was performed by clinical examination based on standard WHO developmental milestones and the Indian Academy of Pediatrics (IAP) guidelines. Developmental delay was defined as the failure to achieve age‐appropriate milestones in any of the four domains (gross motor, fine motor, social, and language), while neuroregression was specifically documented as the loss of previously attained milestones (such as head control or social smile). Socioeconomic status was categorized using the modified Kuppuswamy Scale [8], and nutritional status was assessed based on WHO‐recommended growth parameters, specifically weight for length and length for age [9].

### 2.5. Laboratory Investigations and Biochemical Analyses

Hematological and biochemical assessments were performed using standardized methods. Complete blood count (CBC) was processed using a three‐part fully automatic hematology analyzer (ZbiyoInc Fully automatic Hematology Analyzer Z3, Dadukou, Chongqing, China) to determine hemoglobin and mean corpuscular volume (MCV). Serum vitamin B12 was analyzed using an ELISA kit (Siemens Healthcare Diagnostics Inc., NY 10591, USA), with the reference range 246–911 pg/mL. While folic acid levels were not systematically measured for all participants due to resource constraints, clinical assessments and peripheral blood smears were used to evaluate features of megaloblastic anemia. Holo‐TC was analyzed using the chemiluminescence method; levels below 5 pmol/L (Lal Path Lab, New Delhi) were considered to denote severe deficiency. EFAs were profiled using dried blood test kits (Lipomic Laboratory, Lipomic Healthcare Pvt. Ltd., New Delhi) to determine the levels of *ω*‐3, *ω*‐6, and saturated fatty acids (SFAs), and the *ω*‐6:ω‐3 ratio was calculated.

### 2.6. Clinical Management

All patients received standardized treatment for vitamin B12 deficiency and its comorbidities. Therapeutic management included intramuscular or oral vitamin B12 supplementation, packed RBC transfusions for severe anemia, and iron–folic acid supplementation, as clinically indicated. Antibiotics and other nutritional supplements were prescribed on a case‐by‐case basis.

### 2.7. Statistical Analysis

Data were entered into a Microsoft Excel spreadsheet and analyzed using Stata Version 12 (StataCorp, College Station, TX). Continuous variables were assessed for distribution and are presented as medians with interquartile ranges (Q1–Q3), given the skewed nature of the data and small sample size.

The primary analytical approach was based on estimation. Between‐group differences were summarized as effect sizes (difference in group medians) with corresponding 95% confidence intervals to reflect the magnitude and precision of associations. Nonparametric methods (Wilcoxon rank‐sum test) were used to support comparisons where appropriate. Categorical variables and clinical characteristics are presented as frequencies and percentages. For variables with values below the analytical detection limit (e.g., holo‐TC < 5 pmol/L), results are reported descriptively without formal statistical comparison. Where reported, *p* values were interpreted using a two‐sided 5% significance level, although conclusions were drawn primarily from effect sizes and their 95% confidence intervals.

## 3. Results

### 3.1. Demographic and Clinical Profile of the Cohort

The study analyzed a total of 14 infants, divided into two cohorts: Group 1 (ITS with tremors, *n* = 7) and Group 2 (ITS without tremors, *n* = 7) (Figure 1). The mean (±SD) age was 9.5 (±3.9) months in Group 1 and 8.7 (±4.9) months in Group 2, with male preponderance (11 males, 3 females overall) (Supporting Table 1). All subjects belonged to lower socioeconomic categories (scores 3–5 on the modified Kuppuswamy Scale), resided predominantly in rural areas, and were from Hindu families (Supporting Table 1). None had severe acute malnutrition. Developmental delay was prevalent, with severe delay in 71.4% (5/7) of Group 1 infants. Skin hyperpigmentation was more common in Group 1 (71.4%, 5/7) than in Group 2 (42.9%, 3/7). Hair changes were also more frequent in Group 1 (57.1%, 4/7) than in Group 2 (42.9%, 3/7). Regression of milestones occurred in 3 children per group.

### 3.2. Dietary and Feeding Patterns

Feeding histories highlighted nutritional vulnerability. No children in Group 1 received complementary foods at the WHO‐recommended age of 6 months; introduction was typically delayed to 8–12 months (Table 1). Most mothers were vegetarians, with some reporting irregular or insufficient nonvegetarian intake (Table 1).

**TABLE 1:** Demographic, clinical profile, dietary, and feeding profile of children included in the study (*n* = 14).

Age (months)		Sex		Socioeconomic status^∗^		Area		Nutritional status^†^				Pallor		Skin hyperpigmentation		Hair changes	
Group 1	Group 2	Group 1	Group 2	Group 1	Group 2	Group 1	Group 2	Group 1		Group 2		Group 1	Group 2	Group 1	Group 2	Group 1	Group 2
								Weight‐for‐length	Length‐for‐age	Weight‐for‐length	Length‐for‐age						
3.5	3	M	M	4	4	Rural	Urban	<−3 SD	Normal	Normal	Normal	Yes	No	No	No	No	No
7	5	M	F	4	4	Rural	Rural	Normal	Normal	Normal	<−3 SD	Yes	Yes	Yes	Yes	Yes	Yes
7	6	F	M	3	3	Rural	Rural	<−2 SD	<−2 SD	Normal	Normal	Yes	Yes	No	No	No	No
10	7	M	M	3	3	Rural	Urban	<−2 SD	Normal	Normal	Normal	Yes	Yes	Yes	No	No	No
12	11	M	M	4	3	Rural	Rural	Normal	Normal	Normal	Normal	Yes	Yes	Yes	No	Yes	No
12	12	M	M	3	5	Rural	Rural	Normal	<−2 SD	Normal	<−2 SD	Yes	Yes	Yes	Yes	Yes	Yes
15	17	M	F	4	4	Rural	Rural	<−2 SD	<−3 SD	<−2 SD	Normal	Yes	Yes	Yes	Yes	Yes	Yes

^∗^SES was assessed using the modified Kuppuswamy Socioeconomic Status Scale (income criteria updated for the study year).

^†^Nutritional status was classified according to the World Health Organization (WHO) child growth standards.

### 3.3. Hematological and Vitamin B12 Status

All study participants were anemic (hemoglobin < 11 g/dL). Group 1 demonstrated higher macrocytosis, with a median MCV of 98 fL (Q1–Q3: 94.9–110) compared to 75.5 fL (68–92.4) in Group 2, corresponding to a median difference of 22.5 fL (95% CI: 6.8–38.2) (Table 2).

**TABLE 2:** Comparative analysis of results of hematological, vitamin B12 levels, and essential fatty acid levels in children with ITS with tremors (*n* = 7) and ITS without tremors (*n* = 7).

Variable	Group 1 (ITS with tremor) median (Q1–Q3)	Group 2 (ITS without tremor) median (Q1–Q3)	Estimated difference (95% CI)^∗^	*p* value^†^
Hemoglobin (g/dL)	7 (4.7–8.9)	9.5 (7.4–10.3)	−2.5 (−5.8 to 0.8)	0.141
MCV (fL)	98 (94.9–110)	75.5 (68–92.4)	22.5 (6.8 to 38.2)	0.034
Serum vitamin B12 (pg/mL)	188 (168–218)	198 (189–221)	−10 (−42.5 to 22.5)	0.442
Holo‐TC (pmol/L)	< 5 (all)	10.12 (6.4–20.3)	Not estimable^‡^	—
Omega‐3 (%)	2.4 (1.7–3.6)	2.1 (1.2–3.2)	0.3 (−1.2 to 1.8)	0.405
Omega‐6 (%)	26.9 (24.2–27.3)	23.6 (18.1–26.4)	3.3 (−0.5 to 7.1)	0.084
Omega‐9 (%)	21.4 (19.4–26.0)	23.6 (19.7–28.9)	−2.2 (−7.8 to 3.4)	0.949
Saturated FA (%)	45.3 (42.8–47.7)	47 (44.6–51.9)	−1.7 (−6.5 to 3.1)	0.110
Trans FA (%)	2.3 (1.6–3.7)	2.3 (1.9–4.8)	0.0 (−2.1 to 2.1)	0.222
ω‐6:*ω*‐3 ratio	10.1 (8.3–14.5)	9.2 (8.3–18.8)	0.9 (−5.0 to 7.0)	0.898
Palmitic acid index (%)	29.5 (27.9–30.5)	29.2 (26.2–32.7)	0.3 (−4.2 to 4.8)	1.000

*Note:* Effect sizes are reported as differences between group medians. Standard deviations were approximated from interquartile ranges using established methods; therefore, confidence intervals should be interpreted as approximate estimates.

^∗^Values represent estimated between‐group differences (Group 1 − Group 2) with corresponding 95% confidence intervals.

^‡^Effect size for holo‐transcobalamin (holo‐TC) could not be estimated due to uniformly undetectable values in Group 1.

^†^ *p* values were derived from the Wilcoxon rank‐sum test.

Median serum vitamin B12 levels were below the normal threshold (> 300 pg/mL) in both groups: 188 pg/mL (168–218) in Group 1 and 198 pg/mL (189–221) in Group 2. The between‐group difference was −10 pg/mL (95% CI: −42.5 to 22.5), indicating no clear difference between groups (Table 2).

All infants in Group 1 exhibited severe holo‐TC deficiency, with levels below the analytical detection limit (< 5 pmol/L). In contrast, Group 2 had a median holo‐TC level of 10.12 pmol/L (6.4–20.3), although one infant also demonstrated values < 5 pmol/L. As values in Group 1 were uniformly below the detection limit, effect size estimation was not feasible, and results are presented descriptively (Table 2).

### 3.4. EFA Analysis

Serum EFA profiles showed multiple deviations from reference ranges in both groups, with small between‐group differences and wide confidence intervals, suggesting imprecise estimates (Table 2). Median ω‐3 fatty acid levels were low in both groups (Group 1: 2.4% and Group 2: 2.1%), with a difference of 0.3% (95% CI: −1.2 to 1.8). ω‐6 levels were slightly higher in Group 1, with a difference of 3.3% (95% CI: −0.5 to 7.1). Both groups demonstrated elevated SFA levels (> 40%), with a difference of −1.7% (95% CI: −6.5 to 3.1). The ω‐6:ω‐3 ratio was elevated in both groups (Group 1: 10.1 and Group 2: 9.2), with a difference of 0.9 (95% CI: −5.0 to 7.0). Similarly, the palmitic acid index was high in both groups, with a difference of 0.3 (95% CI: −4.2 to 4.8).

## 4. Discussion

In this exploratory study, we evaluated serum holo‐TC and EFAs in infants with ITS, using an estimation‐based approach. Infants with tremors consistently exhibited extremely low holo‐TC levels (< 5 pmol/L), whereas those without tremors had higher but still suboptimal levels. In contrast, between‐group differences in EFAs were small with wide confidence intervals, suggesting imprecise estimates and no clear association with tremor status.

In tropical and resource‐limited regions, the burden of ITS remains significantly underestimated due to overlapping clinical features with other tropical infections and nutritional deficiencies. The clinical implications in these settings are profound, as early recognition of the clinical spectrum and biochemical thresholds can prevent unnecessary, expensive diagnostic workups and target‐driven maternal–fetal nutritional screening. Strengthening public health strategies around maternal vitamin B12 and EFA supplementation is critical to mitigating this regional burden.

The age of all study participants ranged from 3 to 18 months (Table 1). Bajpai et al. reported the age of 134 patients with ITS between 5 and 25 months [10]. The same age group has been reported in other studies conducted in patients with ITS [2, 11]; however, another study reported ITS in children beyond 2 years of age [1]. In the present study, of the seven infants, only one girl child in Group 1 was diagnosed as having ITS; this male preponderance has been documented in literature [10]. All but one patient included in the present study were admitted in the winter season. However, no seasonal predilection has been documented, with cases being reported throughout the year [1]. Additionally, most children in Group 1 exhibited respiratory viral illness before experiencing tremors, consistent with observations in a previous study [1].

The feeding pattern of mothers of the enrolled infants showed a predominance of a vegetarian diet, though some mothers reported having a limited nonvegetarian diet, but not on a regular basis. Maternal vitamin B12 and holo‐TC levels could not be assayed in this study due to limited resources. However, recent evidence highlights that neonatal vitamin B12 status is significantly correlated with maternal levels, suggesting that the clinical manifestations of ITS in early infancy are often a direct consequence of maternal depletion during pregnancy and lactation [12]. Characterizing this maternal–fetal nutritional axis is essential for understanding the origin of ITS, as infants born to deficient mothers have limited hepatic stores to sustain them through the first few months of life [12]. Clinical signs of vitamin B12 deficiency become apparent in infants born to mothers with low vitamin B12 levels within the first 6–18 months of life [7].

None of the infants in either group had severe undernutrition. Moreover, infants with ITS appeared chubby and well‐built with hypopigmented sparse hairs [10]. The hair changes observed in our cohort, including thinning and light‐brown discoloration (hypochromotrichia), are consistent with the chronic nutritional insult seen in ITS. These changes result from impaired melanin synthesis and altered protein metabolism in hair follicles due to severe vitamin B12 deficiency, serving as a visible indicator of the long‐standing nature of the nutritional depletion before the onset of neurological symptoms. This lack of association between ITS and severe malnutrition is consistent with the findings of other studies [1, 11].

Anemia (Hb < 11 gm/dL) was documented in all infants included in the study. Six out of seven patients in Group 1 had an MCV of > 95 fL, suggestive of macrocytosis. On the other hand, only one out of 7 patients in Group 2 had an MCV indicative of macrocytosis, whereas 4 patients had an MCV indicative of microcytosis. Both groups had vitamin B12 deficiency, clinical signs of which were observed in each patient. Sachdeva et. al., through peripheral blood studies, reported that 58 out of 80 patients with ITS had macrocytic anemia [13]. This suggests that peripheral blood studies poorly correlate with megaloblastic anemia in comparison with serum vitamin B12 or holo‐TC levels.

The higher MCV observed in infants with tremors, with a substantial estimated difference and relatively narrow confidence interval, suggests a meaningful difference in macrocytosis between groups. This finding supports the role of vitamin B12 deficiency–related hematological changes in ITS, although overlap between groups indicates variability at the individual level.

Infantile vitamin B12 deficiency may have typical neurological features such as cerebral and optic nerve atrophy, apathy, coma, hypotonia, and developmental delay [14]. Mild‐to‐severe developmental delay was observed in four infants of Group 1 with neuroregression. All these infants were found to have low or normal levels of vitamin B12 and extremely low levels (< 5 pmol/L) of holo‐TC. In Group 2, only 4 out of 8 infants presented with either developmental delay or regression, and vitamin B12 levels in all infants were below the normal range, although only one had extremely low levels of holo‐TC (< 5 pmol/L). This suggests that holo‐TC levels may better reflect the presence of tremors, warranting a larger study to ascertain this relationship. MRI scans have shown severe diffuse cerebral atrophy in patients with ITS [15]. However, follow‐up MRI scans done after 6 months of nutritional rehabilitation demonstrated the reversal of cerebral atrophy, underscoring the nutritional etiology of ITS [15, 16].

The neurological symptoms associated with ITS, such as developmental delays and regression, apathy, and anorexia, are consistent with the known manifestations of vitamin B12 deficiency [17]. Specifically, severe vitamin B12 deficiency in infants can manifest as irritability, failure to thrive, and developmental regression, which typically resolve rapidly with appropriate supplementation [18].

Vitamin B12 has been postulated to be associated with ITS for a long time. Studies from the Indian sub‐continent have described ITS as a vitamin B12 deficiency [1, 18]. In settings constrained by investigation facilities, patients diagnosed as having ITS could be successfully managed with therapeutic doses of vitamin B12 [1]. Recent studies also support the role of vitamin B12 in ITS etiology [11, 15, 19]. Goraya et al. conducted a study in 15 patients with ITS, of whom 13 had vitamin B12 deficiency [19]. However, the role of vitamin B12 deficiency as an etiological factor for ITS remains to be confirmed. While tremors are a hallmark of ITS, recent literature emphasizes that ITS is a clinical spectrum where severe systemic manifestations, including life‐threatening infections, can occur even in the absence of tremors [20]. The classification of infants without tremors into the ITS spectrum in our study is supported by the presence of other hallmark features, such as skin hyperpigmentation and neuroregression. This suggests that tremors may be a threshold‐dependent manifestation of severe biochemical exhaustion of active Vitamin B12. In the present study, we evaluated the role of the active biomarker of vitamin B12, that is, serum holo‐TC, for the first time and identified that all seven ITS cases with tremors (Group 1) had extremely low holo‐TC levels. However, one infant in Group 2 also had a low‐serum holo‐TC level of < 5 pmol/L. Although both groups had low total serum vitamin B12 levels with minimal between‐group differences, holo‐TC levels showed a marked contrast. All infants with tremors had values below the detection limit, whereas most infants without tremors had measurable levels. While effect size estimation was not feasible due to uniformly undetectable values in one group, the observed pattern suggests a potential association between severe bioactive vitamin B12 deficiency and tremor manifestation. However, given the small sample size, this finding should be interpreted cautiously and requires confirmation in larger studies.

The present study also considered the deficiency of serum EFAs as an etiological factor for ITS because complementary feeding in patients with ITS has been reported to be delayed or lacking. In adults, the deficiency of *ω*‐3 fatty acids or an imbalance in the *ω*‐3:*ω*‐6 ratio has been implicated in various neurological disorders, including Alzheimer’s disease, Parkinson’s disease, Huntington’s disease, stroke, multiple sclerosis, and psychotic disorders [7]. Neurons lack the enzymes for the de novo synthesis of the EFAs docosahexaenoic acid (DHA) and arachidonic acid (AA); therefore, acquisition of these fatty acids relies on dietary sources [21]. DHA is essential for the normal functional development of the retina and brain, particularly in premature infants [21]. Inclusion of *ω*‐3 fatty acids has been recommended in the human diet because of their vital role in growth and development throughout the life span of humans [21]. The present study observed an increased *ω*‐6:*ω*‐3 ratio in all patients with ITS (Table 2), irrespective of the presence or absence of tremors. The exploration of EFA profiles in infants with ITS represents a novel dimension of our study. To the best of our knowledge, the association between EFA levels and the clinical manifestations of ITS remains hitherto unexplored in existing literature. While previous research has extensively documented the role of Vitamin B12 deficiency in ITS, the potential contribution of EFA—particularly the balance between ω‐6 and ω‐3 fatty acids—to neurodevelopmental outcomes in these infants has not been systematically investigated. This finding indicates that an imbalance in the *ω*‐6:ω‐3 ratio, rather than an absolute deficiency, may contribute to the neurological manifestations observed in ITS, although the precise mechanisms warrant further investigation. Furthermore, inadequate levels of EFAs can negatively influence neurocognitive development in infants, either due to their direct association with structural impairments in the brain or indirect effects on motor behavior and overall experience [22]. Inconsistencies in the findings regarding vitamin and fatty acid deficiencies in children with autism spectrum disorders further underscore the need for comprehensive research on their precise roles in neurodevelopmental conditions [23]. Similarly, deficiencies in various micro‐ and macronutrients, including vitamins, are known to influence neurodevelopment because of their role as cofactors in numerous biochemical pathways [24].

Both groups demonstrated abnormalities in EFA profiles, including low ω‐3 levels, elevated SFAs, and increased *ω*‐6:ω‐3 ratios. However, differences between groups were small and accompanied by wide confidence intervals, limiting the precision of these estimates. These findings suggest that while EFA imbalance may be common in ITS, its specific role in tremor manifestation remains uncertain. Larger studies are needed to clarify whether these observed patterns represent true associations or reflect underlying nutritional vulnerability. However, the observed abnormal ratio of *ω*‐6:ω‐3 fatty acids and high levels of SFAs in the study participants raise concerns regarding health of these children in the long term because the intake of large amounts of *ω*‐6 fatty acids shifts the physiologic state to one that is prothrombotic and proaggregatory, characterized by increases in blood viscosity, vasospasm, and vasoconstriction and a decrease in bleeding time; these alterations may lead to an increased risk of various noncommunicable diseases [7].

A critical appraisal of our findings suggests that while total vitamin B12 levels were low in both groups, the absolute depletion of holo‐TC in the tremor group is the most significant biochemical differentiator. This implies that tremors in ITS may not just be a result of overall B12 deficiency, but a marker of cellular‐level metabolic exhaustion of the active vitamin fraction. Furthermore, the persistent EFA imbalance across both groups indicates a baseline nutritional vulnerability in this population, which may lower the threshold for neurological symptoms when combined with acute B12 depletion.

A key strength of this study is the use of an estimation‐based analytical approach, emphasizing effect sizes and confidence intervals, which provides a more informative interpretation of findings in small sample exploratory research. The small sample size limits the precision of effect estimates, as reflected by wide confidence intervals for several comparisons. Consequently, the findings should be interpreted as exploratory, and the absence of clear differences should not be interpreted as evidence of no association. A limitation of this pilot study is the lack of folic acid level estimations. Although megaloblastic anemia in the Indian subcontinent is often multifactorial, the rapid clinical improvement observed after vitamin B12 administration in previous studies and the extremely low holo‐TC levels in our cohort strongly point toward B12 deficiency as the primary driver of tremors in these infants. Furthermore, establishing clear causal links between specific nutritional factors and complex outcomes is complicated by the co‐occurrence of multiple nutrient deficiencies and a myriad of nonnutritional confounding variables, such as socioeconomic status and environmental stressors.

## 5. Conclusion

This exploratory comparative study highlights a consistent pattern of extremely low holo‐TC levels in infants with ITS presenting with tremors, suggesting a potential association between severe bioactive vitamin B12 deficiency and neurological manifestations. In contrast, EFA abnormalities were observed in both groups, with small and imprecise between‐group differences, limiting conclusions regarding their role in tremor occurrence.

Given the small sample size and limited precision of estimates, these findings should be interpreted cautiously. Larger, well‐designed studies are needed to confirm the role of holo‐TC as a clinically relevant biomarker and to further explore the contribution of EFA imbalances in the pathophysiology of ITS.

## Author Contributions

Rajesh Gupta: conceptualization, data collection and analysis, drafting manuscript, and revision; Puneet Agrawal: data collection, analysis, and manuscript drafting; Jyoti Singh and Jyoti Gupta: critical inputs and manuscript drafting; and Ashish Pathak: data interpretation and analysis, critical inputs, drafting manuscript, and revision.

## Funding

The authors have nothing to report.

## Disclosure

All authors approved the final manuscript and are accountable.

## Conflicts of Interest

The authors declare no conflicts of interest.

## Supporting Information

Additional supporting information can be found online in the Supporting Information section.

## Supporting information


**Supporting Information 1** Supporting Table 1: Demographic and clinical characteristics of the study participants (*n* = 14). ^∗^Values are represented as median [range] for biochemical parameters and mean ± SD for hematological parameters.

**Supporting Information 2** STROBE_ITS.

## Data Availability

The data that support the findings of this study are available on request from the corresponding author. The data are not publicly available due to privacy or ethical restrictions.

## References

[1] Gupta R. , Rawat A. K. , Singh P. , Gupta J. , and Pathak A. , Infantile Tremor Syndrome: Current Perspectives, Research and Reports in Tropical Medicine. (July 2019) 10, 103–108, 10.2147/rrtm.s180604. 31308787 PMC6615725

[2] Goraya J. S. and Kaur S. , Infantile Tremor Syndrome–down but Not Out, Indian Pediatrics. (2015) 52, 249–250. 25849009 10.1007/s13312-015-0619-9

[3] Hannibal L. , Lysne V. , Bjørke-Monsen A. L. et al., Biomarkers and Algorithms for the Diagnosis of Vitamin B12 Deficiency, Frontiers in Molecular Biosciences. (May 2016) 3, 10.3389/fmolb.2016.00027. PMC4921487 27446930

[4] von Schenck U. , Bender-Götze C. , and Koletzko B. , Persistence of Neurological Damage Induced by Dietary Vitamin B-12 Deficiency in Infancy, Archives of Disease in Childhood. (August 1997) 77, no. 2, 137–139, 10.1136/adc.77.2.137. 9301352 PMC1717263

[5] Naik S. , Mahalle N. , and Bhide V. , Identification of Vitamin B12 Deficiency in Vegetarian Indians, British Journal of Nutrition. (March 2018) 119, no. 6, 629–635, 10.1017/S0007114518000090. 29446340

[6] von Castel-Roberts K. M. , Morkbak A. L. , Nexo E. et al., Holo-Transcobalamin is an Indicator of Vitamin B-12 Absorption in Healthy Adults With Adequate Vitamin B-12 Status, American Journal of Clinical Nutrition. (April 2007) 85, no. 4, 1057–1061, 10.1093/ajcn/85.4.1057. 17413105

[7] Saini S. K. and Keum Y. S. , Omega-3 and Omega-6 Polyunsaturated Fatty Acids: Dietary Sources, Metabolism, and Significance–A Review, Life Sciences. (2018) 203, 255–267, 10.1016/j.lfs.2018.04.049. 29715470

[8] Radhakrishnan M. and Nagaraja S. B. , Modified Kuppuswamy Socioeconomic Scale 2023: Stratification and Updates, International Journal of Community Medicine and Public Health. (2023) 10, no. 11, 4415–4418, 10.18203/2394-6040.ijcmph20233487.

[9] World Health Organization , WHO Child Growth Standards: Length/Height-for-Age, Weight-for-Age, Weight-for-Length, Weight-for-Height and Body Mass Index-for-Age, Methods and Development. (2006) https://www.who.int/publications/i/item/924154693X.

[10] Bajpai P. C. , Misra P. K. , and Tandon P. N. , Further Observations on Infantile Tremor Syndrome, Indian Pediatrics. (1968) 5, 297–307. 5711417

[11] Gehlot P. , Gupta R. , Mandliya J. C. , Singh P. , and Pathak A. , Cranial Neuroimaging in Infantile Tremor Syndrome: The Road Ahead, Journal of Clinical and Diagnostic Research. (March 2018) 12, no. Issue 3, SC01–SC04, 10.7860/JCDR/2018/29522.11240.

[12] Ferraro S. , Lucchi S. , Montanari C. et al., Vitamin B12 Deficiency in Newborns: Impact on Individual’s Health Status and Healthcare Costs, Clinical Chemistry and Laboratory Medicine. (2025) 63, no. 3, 559–571, 10.1515/cclm-2024-0692. 39356629

[13] Sachdev K. K. , Manchanda S. S. , and Lal H. , The Syndrome of Tremors, Mental Regression and Anaemia in Infants and Young Children: A Study of 102 Cases, Indian Pediatrics. (1965) 2, no. 7, 239–251. 5834538

[14] Stollhoff K. and Schulte F. J. , Vitamin B12 and Brain Development, European Journal of Pediatrics. (1987) 146, no. 2, 201–205, 10.1007/BF02343237. 3569363

[15] Gupta R. , Pathak A. , Mandliya J. , Mandliya P. , and Sonker P. , Reversible Cerebral Atrophy in Infantile Tremor Syndrome, Indian Pediatrics. (August 2016) 53, no. Issue 8, 727–729, 10.1007/s13312-016-0918-9. 27395831

[16] Gunston G. D. , Burkimsher D. , Malan H. , and Sive A. A. , Reversible Cerebral Shrinkage in Kwashiorkor: An MRI Study, Archives of Disease in Childhood. (August 1992) 67, no. Issue 8, 1030–1032, 10.1136/adc.67.8.1030. 1520007 PMC1793595

[17] Nawaz A. , Khattak N. N. , Khan M. S. , Nangyal H. , Sabri S. , and Shakir M. , Deficiency of Vitamin B12 and Its Relation With Neurological Disorders: A Critical Review, Journal of Basic and Applied Zoology. (March 2020) 81, no. 1, 10.1186/s41936-020-00148-0.

[18] Balabanović M. and Shoham Y. , Fab: Content-Based, Collaborative Recommendation, Communications of the ACM. (1997) 40, no. 3, 66–72, 10.1145/245108.245124.

[19] Goraya J. S. , Acute Movement Disorders in Children: Experience From a Developing Country, Journal of Child Neurology. (2015) 30, no. 4, 406–411, 10.1177/0883073814550828. 25296919

[20] Nandi M. , Gupta N. , and Roy P. , Infantile Tremor Syndrome: Time for ‘Rethink’ and a ‘Relook, Medical Journal of Dr. D.Y. Patil Vidyapeeth. (2026) 19, no. 2, 182–185, 10.4103/mjdrdypu.mjdrdypu_1247_24.

[21] Simopoulos A. P. , Omega-3 Fatty Acids in Health and Disease and in Growth and Development, American Journal of Clinical Nutrition. (1991) 54, no. 3, 438–463, 10.1093/ajcn/54.3.438. 1908631

[22] Cakir M. , Senyuva S. , Kul S. , Sag E. , Cansu A. , Yucesan F. B. , Yaman S. O. , and Orem A. , Neurocognitive Functions in Infants With Malnutrition; Relation With Long-Chain Polyunsaturated Fatty Acids, Micronutrients Levels and Magnetic Resonance Spectroscopy, Pediatric Gastroenterology, Hepatology & Nutrition. (March 2019) 22, no. Issue 2, 171–180, 10.5223/pghn.2019.22.2.171. PMC6416383 30899693

[23] Gallardo-Carrasco M. C. , Jiménez-Barbero J. A. , Bravo-Pastor M. D. M. , Martin-Castillo D. , and Sánchez-Muñoz M. , Serum Vitamin D, Folate and Fatty Acid Levels in Children With Autism Spectrum Disorders: A Systematic Review and Meta-Analysis, Journal of Autism and Developmental Disorders. (November 2022) 52, no. Issue 11, 4708–4721, 10.1007/s10803-021-05335-8. 34734376 PMC9556366

[24] Hosseini S. M. , Panahi-Azar A. , Sheybani-Arani M. et al., Vitamins, Minerals and Their Maternal Levels’ Role in Brain Development: An Updated Literature-Review, Clinical Nutrition ESPEN. (October 2024) 63, 31–45, 10.1016/j.clnesp.2024.05.011. 38907995

