# Supplementary material for: Unveiling Nutritional Disparities in Infantile Tremor Syndrome: A Focus on Holo‐Transcobalamin and Essential Fatty Acids
Source: J Trop Med. 2026 Jul 18;2026:3144439. doi: 10.1155/jotm/3144439 (PMC13379889; doi:10.1155/jotm/3144439)
Supplement: Supplementary file 1 — Supporting Information 1 Supporting Table 1: Demographic and clinical characteristics of the study participants (n = 14). ∗Values are represented as median [range] for biochemical parameters and mean ± SD for hematological parameters. [file JOTM-2026-3144439-s001.docx]

**Supplementary Table 1: Demographic and Clinical Characteristics of the Study Participants (n=14)**

| **Parameters** | **Group 1 (ITS with tremors) (n=7)** | **Group 2 (ITS without tremors) (n=7)** |
| --- | --- | --- |
| **Demographics** |  |  |
| Age in months (Mean ± SD) | 9.5 ± 3.9 | 8.7 ± 4.9 |
| Gender (Male / Female) | 5 / 2 | 6 / 1 |
| Socioeconomic Status | Lower (Kuppuswamy 3–5) | Lower (Kuppuswamy 3–5) |
| Residence | Rural | Rural |
| **Clinical Features** |  |  |
| Tremors | 7 (100%) | 0 (0%) |
| Developmental Delay (Overall) | 7 (100%) | 7 (100%) |
| Severe Developmental Delay | 5 (71.4%) | Not Specified |
| Skin Hyperpigmentation | 5 (71.4%) | 3 (42.9%) |
| Hair Changes | 4 (57.1%) | 3 (42.9%) |
| Regression of Milestones | 3 (42.9%) | 3 (42.9%) |
| Severe Acute Malnutrition | 0 (0%) | 0 (0%) |
| **Laboratory Parameters** |  |  |
| Serum Vitamin B12 (pg/mL) | 142.5 [98–180]* | 165.0 [110–210]* |
| **Serum Holo-TC (pmol/L)** | **<5 (Undetectable)** | **12.4 [8.5–18.2]*** |
| Hemoglobin (g/dL) | 7.2 ± 1.1* | 7.8 ± 0.9* |

**Values are represented as Median [Range] for biochemical parameters and Mean ± SD for hematological parameters.*
