# Supplementary material for: Unveiling Nutritional Disparities in Infantile Tremor Syndrome: A Focus on Holo‐Transcobalamin and Essential Fatty Acids
Source: J Trop Med. 2026 Jul 18;2026:3144439. doi: 10.1155/jotm/3144439 (PMC13379889; doi:10.1155/jotm/3144439)
Supplement: Supplementary file 2 — Supporting Information 2 STROBE_ITS. [file JOTM-2026-3144439-s002.docx]

| **Section** | **Item No.** | **Recommendation** | **Reported in Manuscript** |
| --- | --- | --- | --- |
| **Title and Abstract** | 1a | Indicate study design in title/abstract | Yes – described as comparative pilot study |
|  | 1b | Provide informative and balanced abstract | Yes – structured abstract with background, methods, results, conclusion |
| **Introduction** | 2 | Explain scientific background and rationale | Yes – ITS, vitamin B12, holo-TC, EFAs described |
|  | 3 | State specific objectives/hypotheses | Yes – role of holo-TC and EFAs |
| **Methods** | 4 | Present key elements of study design | Yes – comparative cross-sectional pilot study |
|  | 5 | Describe setting, locations, relevant dates | Yes – GMC Datia, Dec 2023–Apr 2024 |
|  | 6a | Eligibility criteria, sources, selection methods | Yes – clinical ITS criteria, exclusion criteria |
|  | 6b | Matching criteria (if applicable) | Not applicable |
|  | 7 | Define outcomes, exposures, predictors | Yes – holo-TC, EFAs, vitamin B12, clinical variables |
|  | 8 | Data sources/measurement methods | Yes – ELISA, chemiluminescence, hematology analyzer |
|  | 9 | Address potential sources of bias | Partially – discussed in limitations |
|  | 10 | Explain study size | Yes – pilot study + post-hoc power |
|  | 11 | Handling of quantitative variables | Yes – median (IQR), categorical percentages |
|  | 12a | Statistical methods used | Yes – Mann–Whitney U test |
|  | 12b | Methods for subgroups/interactions | Not applicable |
|  | 12c | Handling of missing data | Not explicitly stated |
|  | 12d | Address loss to follow-up | Not applicable (cross-sectional) |
|  | 12e | Sensitivity analyses | Not performed |
| **Results** | 13a | Number of participants at each stage | Yes – 14 enrolled (7 per group) |
|  | 13b | Reasons for non-participation | Not applicable |
|  | 13c | Flow diagram | Yes – Figure 1 |
|  | 14a | Descriptive data of participants | Yes – demographics, SES, clinical features |
|  | 14b | Missing data | Not explicitly stated |
|  | 14c | Follow-up time | Not applicable |
|  | 15 | Outcome data | Yes – hematological, biochemical, EFA results |
|  | 16a | Main results with estimates and precision | Yes – medians, IQR, p-values |
|  | 16b | Category boundaries reported | Yes – reference ranges provided |
|  | 16c | Translate relative to absolute risk | Not applicable |
|  | 17 | Other analyses | Yes – post-hoc power analysis |
| **Discussion** | 18 | Summarize key results | Yes |
|  | 19 | Discuss limitations | Yes – small sample, confounding |
|  | 20 | Provide interpretation | Yes – holo-TC significance, EFA role |
|  | 21 | Generalisability | Yes – limited generalizability acknowledged |
| **Other Information** | 22 | Funding and role of funders | Yes – no funding declared |
